# Description of new species of Trematoda from bats of Southeastern Mexico and a new classification for *Brachylecithum rileyi* n. comb. (Dicrocoeliidae)

**DOI:** 10.1007/s11230-023-10127-y

**Published:** 2023-12-18

**Authors:** Wilson I. Moguel-Chin, Jesús Alonso Panti-May, Brenda Atziri García-García, David I. Hernández-Mena

**Affiliations:** 1 Facultad de Medicina Veterinaria y Zootecnia, Universidad Autónoma de Yucatán, Campus de Ciencias Biológicas y Agropecuarias, km 15.5 carretera Mérida-Xmatkuil, 97135 Mérida, Yucatán Mexico; 2 Centro de Investigaciones Regionales ‘Dr. Hideyo Noguchi’, Universidad Autónoma de Yucatán, Av. Itzáes, Centro, 97000 Mérida, Yucatán Mexico; 3 Instituto de Biología, Universidad Nacional Autónoma de Mexico, Avenida Universidad 3000, CU, 04510 Coyoacán, CDMX Mexico; 4 Centro de Investigación y de Estudios Avanzados, Instituto Politécnico Nacional, Unidad Mérida, Carretera Mérida-Progreso, Loma Bonita, 97205 Mérida, Yucatán Mexico; 5 Escuela Nacional de Estudios Superiores, Universidad Nacional Autónoma de Mexico, Carretera Mérida-Tetiz Km4, 97357 Ucú, Yucatán Mexico

## Abstract

To date, 23 species of trematodes have been reported in bats from Mexico. However, in some regions of Mexico, such as the Yucatan Peninsula, many species of bats do not have helminthological records. Here, we sampled bats in four localities in Southeastern Mexico from April 2017 to February 2022. Parasites were collected from the intestine of four species of bats: *Pteronotus fulvus*, *Eumops nanus*, *Noctilio leporinus* and *Nyctinomops laticaudatus*. Conventional morphological techniques and molecular tools with the 28S ribosomal gene were used to describe the helminths. We discovered that our parasites represent three new species and a new combination of trematodes. We found that the new species have morphological differences with their congeneric species, and we complement this information whit molecular data. Furthermore, we found morphological and molecular evidence that places *Dicrocoelium rileyi* within the genus *Brachylecithum*. This study points out the importance of comparing morphological and phylogenetic information.

**Supplementary Information:**

The online version contains supplementary material available at 10.1007/s11230-023-10127-y.

## Introduction

In Mexico, more than 50 species of helminths have been recorded in bats, of which 23 are trematodes (Caspeta-Mandujano et al., 2017; Panti-May et al., 2021; Moguel-Chin et al., 2023) Probably in this group of mammals it is still possible to find a wide variety of new species of trematodes (e. g. Fernandes et al., 2019; Cacique et al., 2023), because the records are based on morphology (Salinas-Ramos et al., 2017). However, the use of molecular techniques and phylogenetic analyzes have proven to be powerful methods to detect new species and separate species that are difficult to differentiate with morphological characters (Hernández-Mena et al., 2022). Particularly, in regions such as the Yucatan Peninsula, many species of bats do not have helminthological records and most of them have been reported recently (Moguel-Chin et al., 2023). In previous studies (Panti-May et al., 2021; Moguel-Chin et al., 2023), where the helminthic fauna of several species of bats from the Yucatan Peninsula was recorded through morphological and molecular analysis with DNA sequences, two new species of *Limatulum* Travassos, 1921 and one new species of *Pygidiopsis* Looss, 1907 were found, but they were not described due to the objectives of those investigations. Furthermore, in Moguel-Chin et al. (2023) *Dicrocoelium rileyi* Macy, 1931 was also recorded, a species that, based on morphological observations, has been suggested to belong to *Brachylecithum* Shtrom, 1940. To date, eight species have been described for the genus *Limatulum*, and only five have been recorded from Mexico in six different families of bats (Phyllostomidae, Vespertillionidea, Emballonuridea, Molossidae, Natalidae and Mormopidae): *L*. *aberrans* Caballero and Bravo, 1950; *L*. *diminutum* Chandler, 1938; *L*. *gastroides* Macy, 1935; *L*. *limatulum* Braun, 1900, and *L*. *oklahomense* Macy, 1931 (Salinas-Ramos et al., 2017; Caspeta-Mandujano et al., 2017). On the other hand, the genus *Pygidiopsis* includes 14 species (Sohn et al., 2016) but only *P*. *macrostumum* Looss, 1907 has been reported in Mexico (Panti-May et al., 2021; Moguel-Chin et al., 2023). However, Moguel-Chin et al. (2023) observed that some morphological characters of the specimens recorded in Mexico differ from the specimens of *P*. *macrostumum* described by Simões et al. (2005). In addition, the species *D. rileyi* had also already been recorded in the country (e.g. Caspeta-Mandujano et al., 2017; Martínez-Salazar et al., 2020). Therefore, the objectives of this study are to describe three new species of trematodes, two from *Limatulum* and one from *Pygidiopsis*, propose a formal taxonomic change from *D. rileyi* to *Brachylecithum* and explore the phylogenetic relationship of these helminths with DNA sequences.

## Methods

### Specimen collection

In the Yucatan Peninsula, the bats *Eumops nanus* (Miller), *Nyctinomops laticaudatus* (Geoffroy), *Pteronotus fulvus* (Thomas) and *Noctilio leporinus* (Linnaeus) were collected in three sites from May 2017 to February 2021, and in Veracruz *P*. *fulvus* was collected in April 2017, under permits from the Mexican Ministry of Environment (SGPA/DGVS/03705/17 and SGPA/DGVS/00786/21; FAUT-0170 and FAUT-0056 respectively). Bats were captured using mist nets (12 m wide × 2.5 m high) for one night, placed in cloth bags, and identified following Medellín et al. (2008). Bats were anesthetized with isoflurane and euthanized by overdose of sodium pentobarbital. The heart, lungs, stomach, liver, small and large intestines, and mesenteries of each specimen were collected and stored in 96% ethanol. All collected organs were dissected from each bat and immersed in distilled water in Petri dishes using a stereo microscope (Olympus SZ2-ILST). Helminths were collected, counted and preserved in 70% ethanol until morphological and molecular identification. Unlike the helminths collected in Yucatan, the helminths in Veracruz were collected immediately after sacrificing and dissecting the bats, so the trematodes were still alive and were killed with 4% hot formaldehyde for morphological study and others were placed directly in absolute ethanol for molecular study. Bats from the Yucatan Peninsula were deposited in the Colección Zoológica (CZ), Campus de Ciencias Biológicas y Agropecuarias, Universidad Autónoma de Yucatán (Supplementary 1), while bats from Veracruz were deposited in the Colección de Mamíferos del Museo de Zoología “Alfonso L. Herrera”, Facultad de Ciencias, Universidad Nacional Autónoma de Mexico.

### Morphological analysis

All the trematodes destined for the morphological study were stained with carmine acid or Gomori trichrome, dehydrated through an ethanol series, cleared in methyl salicylate, and permanently mounted in Canada balsam. Specimens were studied under light microscopy (Leica DM500). The measurements are presented in micrometres (μm) with the range followed by the mean in parentheses. Trematodes were identified to genus level using the taxonomic key of Bray et al. (2008). For identification and comparison at the species level, the original descriptions of the respective articles were used. Specimens were deposited in the Colección Nacional de Helmintos (CNHE), Universidad Nacional Autónoma de Mexico.

### DNA sequencing and phylogenetic analyses

The procedures for extraction, amplification and DNA sequencing of trematodes were detailed in a previous study (Moguel-Chin et al., 2023). We amplified the 28S gene of ribosomal DNA with the forward primer 391 5′ –AGCGGAGGAAAAGAAACTAA– 3′ (Stock et al., 2001) and the reverse primer 536 5′ –CAGCTATCCTGAGGGAAAC– 3′ (Stock et al., 2001) which amplify a fragment of approximately 1200 base pairs (bp). The obtained sequences were deposited in the Genbank. For the phylogenetic analyses, the new sequences were aligned with those of sequenced species that are available in the Genbank (access numbers in the trees) (Supplementary 2). The phylogenetic method used was Maximum Likelihood (ML) and was executed with 1,000 Bootstrap repetitions to obtain support values for the clades. The phylogenetic analysis procedure is also described in greater detail in Moguel-Chin et al. (2023).

## Results

**Phaneropsolidae Mehra, 1935**

***Limatulum*** **Travassos, 1921**

***Limatulum fulvum* n**. **sp**.

Taxonomic summary

Type-host: *Pteronotus fulvus* (Thomas) (Chiroptera: Molossidae).

Type-locality: Calcehtok (20°33’02.5’’N, 89°54’44.4’’W) Opichén, Yucatan, Mexico.

Other localities: Cueva de los murciélagos (18°03’10.5’’N 95°02’45.1’’W) Sontecomapan, Veracruz, Mexico.

Prevalence and mean intensity: 50% (4/8 bats) and 8.5 from Calcehtok. 60% (3/5 bats) and 2.6 from Sontecomapan.

Material examined: Holotype (CNHE 11747); paratypes: (CNHE 11748, 11749).

Representative DNA sequence: OP837307, OR656691, OR656692, OR656693, OR656694.

Synonym: *Limatulum* **n**. **sp**. 1 in Moguel-Chin et al. (2023)

ZooBank Life Science Identifier: 94FCC493-C98B-4E12-B3FC-183F988856A0

Etymology: The specific name of the new species refers to the name of the species of bat that is the host of this parasite.

### Description

**Fig. 1:**
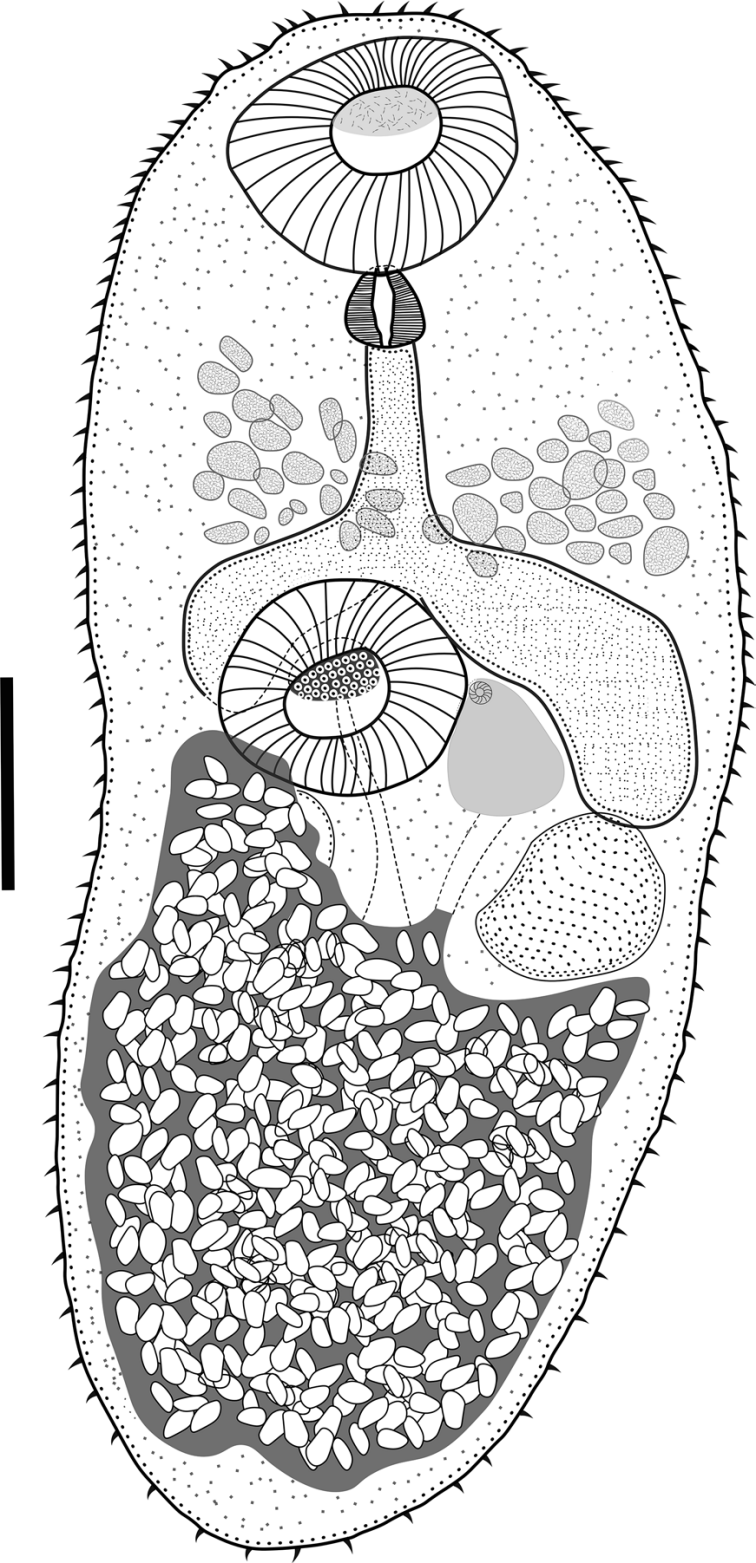
*Limatulum fulvum* **n**. **sp**. ventral view of holotype. The scale bar=100 μm

Based on eight adult specimens (Fig. 1). Body small oval. Body length 500–793 (671), narrow at the oral sucker level, maximum width 225–460 (290) at ventral sucker level. Tegument covered with short, regular spines, diminishing in the posterior half of the body. Oral sucker subterminal and round, 100–141 (118) × 80–136.6 (112.8). Ventral sucker muscular, median, slightly smaller than the oral sucker, 80–126.8 (105.3) × 82.9–122 (103.1). Ventral **/** oral sucker length ratio 1:0.7–1 (1:0.8). Ventral **/** oral sucker width ratio 1:0.6–1.3 (1:0.9). Pharynx muscular oval, 27–55 (36) × 30–36.9 (33.1). Oesophagus long, 24.4–75 (50) long. Intestinal bifurcation anterior to ventral sucker. The caeca short and wide, extending preferentially to the first half to the ventral sucker. Testes rounded, symmetrical, posterior to ventral sucker. Right testis 86.9–110.7 (96.9) × 60–115 (77.8), left testis 90–92 (91) × 68–70 (69). Genital pore sinistral, at the level of the middle of the ventral sucker. Cirrus-sac muscular, small, sinistral of the ventral sucker, 65.3–100 (85.6) × 51.1–70.9 (58.1), containing a long seminal vesicle. The cirrus not observed in any of the specimens. Ovary rounded, pre-testicular, in middle region of the body among the caeca and ventral sucker, 25–89 (57.6) × 25.5–87.3 (61.7). Seminal receptacle and Laurer’s canal not seen. Vitellarium follicular, confluent, in forebody, between the pharynx and the anterior border of the caeca. The uterus covers the posterior third of the body, reaching the anterior region of the testes. Eggs numerous and operculated, 18–23 (19.4) × 7.5–10.5 (9.2). Excretory vesicle Y shaped, excretory pore terminal (see Table 1).

**Table 1:** Morphometric measurements of the species of the genus *Limatulum* described in this study

Species	*L*. *fulvum* **n**. **sp**.	*L*. *fulvum* **n**. **sp**.	*L*. *nanum* **n**. **sp**.	*L*. *aberrans*	*L. mcdanieli*	*L. umbilicatum*	*L*. *diminutum*
Reference	Present study	Present study	Present study	Caballero and Bravo 1950	Brooks and Coggins, 1983	Lunaschi et al. 2003	Caspeta-Mandujano et al. 2017
Type host	*Pteronotus fulvus*	*Pteronotus fulvus*	*Eumops nanus*	*Macrotus waterhousii*	*Myotis lucifungus*	*Myotis nigricans*	*Nycticeius humeralis* and others
Locaties	Yucatan, Mexico	Veracruz, Mexico	Campeche, Mexico	Oaxaca, Mexico	Wisconsin, USA.	Formosa Province, Argentina	Morelos, Mexico
Body length	650–793	500–600	611–745.5	680–708	666–888	557–586	650–820
Body width	225–460	270–310	215.6–393.9	425	366–444	365–370	270–420
Oral sucker	107.3–141 × 104.9–136.6	100–109 × 80–110	122–156.1 × 92.7-161.4	106–111 × 119	98 × 123	131 × 166–193	120–140 × 120–150
Ventral sucker	100–126.8 × 82.9–122	80–98 × 100–110	107.3–151.2 × 92.2–151.2	98 × 98–112	86–111 × 91–123	115–138 × 126–145	110–140 × 120–140
Pharynx	27–55 × 30–36.9	27–40 × 34	24.4–82.9 × 48–70	29–37	37–42 × 37–54	46–55 × 45–56	36 × 51
Oesophagus	24.4–75	56	Short*	49**	86–111	Short*	Short*
Ovary	25–73.2 × 25.5–87.3	61–89 × 65–80	48–61.5 × 43.9–66.9	102–123	51 × 86	68–92 × 64–80	56–75 × 60–83
Right testis	86.9–110.7 × 60–115	90×65	100–122.2 × 65.3–92.7	180 × 106–110	49–123 × 49–123	78–94 × 108–145	100–120 × 70–130
Left testis	92 × 68	90×70	87.7–127.9 × 53.6–93.4	110–115 × 135	49–123 × 49–123	92–97 × 113–126	96–140 × 70–120
Cirrus-sac	65.3–100 × 51.1–70.9	80–100 × 54–55	96–110.7 × 52.8–61.5	172–176 × 74	135–212 × 98–123	216–225 × 69–78	140–220 × 60–80
Eggs	18–23 × 8–10.4	18–20 × 7.5–10.5	19.6–22.1 × 6.6–11.7	20 × 8–10	16–20 × 6–9	17–19 × 10–11	18–23 × 8–15

Species	*L. gastroides*	*L. limatulum*	*L*. *oklahomense*
Reference	Caspeta-Mandujano et al. 2017	Caspeta-Mandujano et al. 2017	Caspeta-Mandujano et al. 2017
Type host	*Balantiopteryx plicata* and others	*Pteronotus mesoamericanus* and others.	*Tadarida cynocephala* and others.
Locaties	Morelos and Jalisco, Mexico	Morelos, Mexico	Morelos, Mexico
Body length	520–570	770–870	690
Body width	290–360	370–450	470
Oral sucker	120–140 × 100–130	100–190 × 170–210	140 × 150
Ventral sucker	110–120 × 100–110	140–170 × 170–200	120 × 140
Pharynx	37–43 × 31–40	65–68 × 60–62	43 × 46
Oesophagus	Short*	Short*	Short*
Ovary	50–60 × 75–87	NA	56×70
Right testis	93–112 × 75–96	85–100 × 100–110	118×112
Left testis	87–100 × 75–87	80–85 × 75–100	100×110
Cirrus-sac	200–220 × 41–43	150–170 × 60–110	130 ×80
Eggs	18–25 × 11–13	15–18 × 8–11	20×11

NA: not available

*No measurements were presented in the description

**In the original description there are no official measurements of the structure, but this measurement was calculated thanks to the scale and the diagram

### Remarks

*Limatulum fulvum* **n**. **sp**. conforms to the diagnosis of *Limatulum* as given by Travassos (1921) by presenting some characteristics such as tegument spinous, testes at or near the level ventral sucker, ovary anterior to the testes and vitellarium in the forebody. The new species is characterized by having the smallest cirrus sac of all species of the genus (Table 1). This new species is also characterized by having a long oesophagus, a character shared with *L. mcdanieli* Brooks & Coggins 1983 and *L. aberrans* Caballero & Bravo-Hollis, 1950 but that differentiates it from the rest of the species. *Limatulum fulvum* **n**. **sp**. can be readily distinguished from *L*. *mcdanieli* by having smaller oesophagus (24.4–75 vs. 86–111); in the new species the vitelline glands are confluent whereas in *L*. *mcdanieli* the vitelline glands are in two clusters; finally, *L*. *mcdanieli* differs from *L*. *fulvum* **n**. **sp**. by exhibiting an anterior fleshy lobe of tissue on the ventral sucker. The new species has an oesophagus similar in size to *L. aberrans*, but differs from this species by presenting a smaller ovary and testes (Table 1); the vitellarium in *L. fulvum* **n**. **sp**. does not reach the pharynx, while in *L. aberrans* does reach the level of the pharynx; finally, the anterior extension of the uterus in *L. fulvum* **n**. **sp**. is up to the anterior region of the testes, while in *L. aberrans* it exceeds the anterior region of said gonads. Other characters that differentiate the new species from its congeners are: the vitelline glands are confluent in *L. fulvum* **n**. **sp**. while in all the others (with the exception of *L. aberrans* and *L. diminutum* Chandler, 1938) they are not confluent and forms two lateral clusters; the vitellarium does not reach the level of the pharynx in the new species, while in all the others (with the exception of *L. mcdanieli*) it does reach the pharynx or the oral sucker; finally, the testes are distributed posteriorly to the ventral sucker, while in *L. diminutum*, *L. gastroides* Macy, 1935, *L. limatulum* Travassos, 1921, *L. ocklahomensi* Macy, 1931 and *L. nanum* **n**. **sp**. they are at the level of this sucker.

***Limatulum nanum* n**. **sp**.

Taxonomic summary

Type-host: *Eumops nanus* (Miller) (Chiroptera: Molossidae).

Type-locality: El Remate (20°30’25.2’’N 90°23’03.0’’W), Calkiní, Campeche, Mexico.

Prevalence and mean intensity: 56 trematodes isolated from an examined bat.

Material examined: Holotype (CNHE 11751); paratypes: (CNHE 11752).

Representative DNA sequence: OP837306

Synonym: *Limatulum* 2 **n**. **sp**. in Moguel-Chin et al. (2023)

ZooBank Life Science Identifier: DE43CC9F-A726-48C6-969C-BB686FEC2AB5

Etymology: The specific name of the new species refers to the name of the host bat.

### Description

**Fig. 2:**
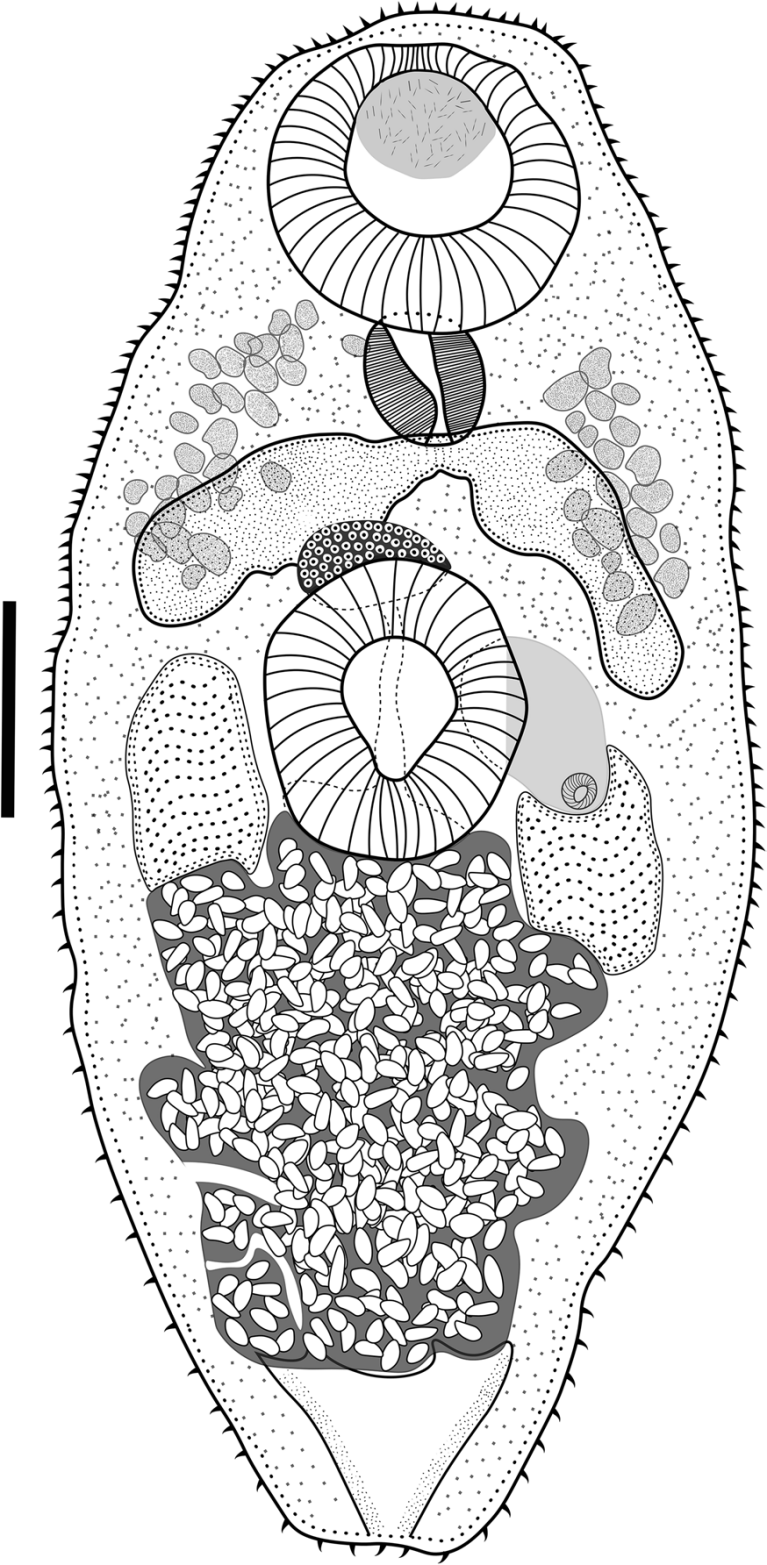
*Limatulum nanum* **n**. **sp**. ventral view of holotype. The scale bar= 100 μm

**Fig. 3:**
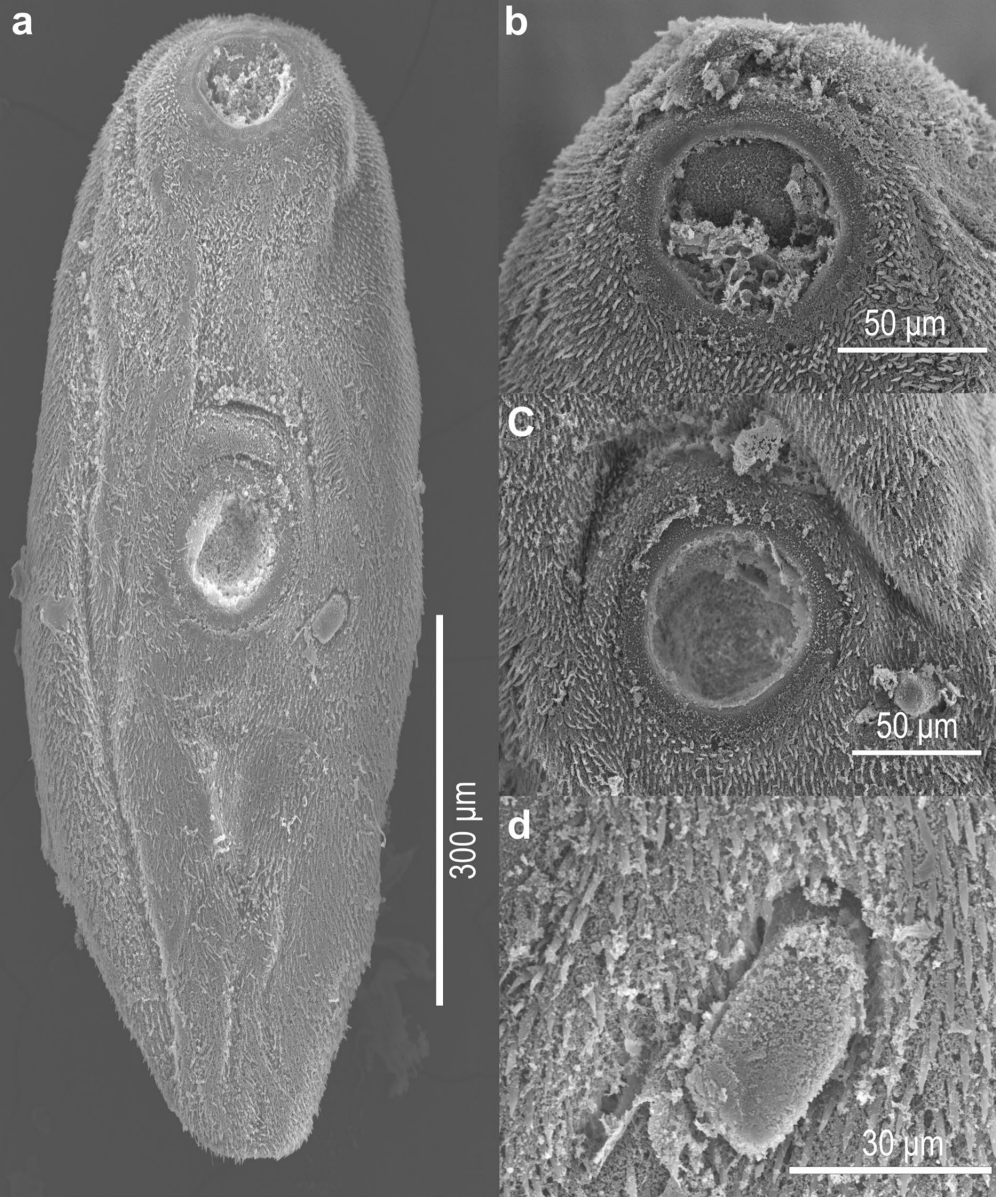
Scanning electron micrographs of *Limatulum nanum* **n**. **sp**. (a) ventral view of the trematode. (b) oral sucker. (c) ventral sucker. (d) external cirrus

Based on 6 adult specimens (Fig. 2, Fig. 3). Body small. Body length 611–745.5 (697.6) narrow at the oral sucker level, width 215.6–393.9 (326.7) at ventral sucker level. Tegument covered entirely by short spines. Oral sucker subterminal and round, 122–156.1 (138.9) × 92.7–161.4 (135.8). Ventral sucker muscular, median, similar in size to oral sucker, 107.3–151.2 (131.7) × 92.2–151.2 (119.9). Ventral /oral sucker length ratio 1:0.7–1 (1:0.9). Ventral / oral sucker width ratio 1:0.6–1 (1:0.8). Pharynx muscular oval, 24–82.9 (54.9) × 48–70 (59.5). Oesophagus absent. Intestinal bifurcation anterior to ventral sucker. The caeca short and wide, extending to the middle of the ventral sucker. Testes rounded, at the level of the ventral sucker zone. Right testis 100–122.2 (110.8) × 65.3–92.7 (78.2), left testis 87.7–127.9 (108.3) × 53.6–93.4 (73.9). Genital pore sinistral, at the level of the posterior edge of the ventral sucker. Cirrus-sac muscular small, on the left testis in the ventral sucker zone and slightly shorter than testes, 96–110.7 (100.8) × 52.8–61.5 (58.1), containing a long seminal vesicle. The cirrus is observed everted, outside the body (Fig. 3c, 3d). Ovary rounded, pre-testicular, between the caeca and the anterior zone of the ventral sucker, 48–61.5 (55.3) × 43.9–66.9 (56.2). Seminal receptacle and Laurer’s canal not observed. Vitellarium follicular, not confluent, in forebody, extending from the posterior border of the oral sucker to the posterior border of the caeca. Uterus in the second half of the body. Eggs numerous and operculated, 19.6–22.1 (20.3) × 6.6–11.7 (8.5). Excretory vesicle Y shaped, excretory pore terminal (see Table 1).

### Remarks

*Limatulum nanum* **n**. **sp**. conforms to the diagnosis of *Limatulum* as given by Travassos (1921) as mentioned above. This new species can be differentiated from its congeners by the size of the cirrus sac: it is larger than the sac of *L. fulvum* **n**. **sp**., but smaller than the rest of the species (Table 1). The new species can be easily differentiated from *L. aberrans*, *L*. *fulvum* **n**. **sp**., *L*. *mcdanieli*, and *L. umbilicatum* Vélez & Thatcher, 1990 because the testes are at the level of the ventral sucker in *L. nanum* **n**. **sp**. while in the aforementioned species the testes are posterior to the ventral sucker. Particularly, the new species differs from *L. umbilicatum* because has oral and ventral suckers similar in size whereas in* L*. *umbilicatum* the oral sucker is wider than the ventral sucker. Additionally, the new species can be distinguished from *L. aberrans*, *L*. *fulvum* **n**. **sp**. and *L*. *mcdanieli* by not having an oesophagus. Particularly, *L*. *mcdanieli* differs from *L*. *nanum* **n**. **sp**. by exhibiting an anterior fleshy lobe on the ventral sucker. *Limatulum nanum* **n**. **sp**., also differs from *L. aberrans*, *L. fulvum* **n**. **sp**. and *L. diminutum* because it has non-confluent vitelline glands grouped in two lateral clusters while the aforementioned species the vitelline glands are confluent. The new species can be distinguished from *L. limatulum* because in *L. nanum* **n**. **sp**. all the tegument is covered by spines and the vitelline glands reach the posterior edge of the ventral sucker whereas in *L. limatulum* the spines extend from the anterior border to the testes and the vitelline glands reach the middle of the ventral sucker. *Limatulum nanum* **n**. **sp**. differs from *L. oklahomense* because in the new species the genital pore is immediately to one side of the ventral sucker and in the middle of this sucker, while in *L. oklahomense* it is lateral to the testis and is posterior to the ventral sucker. Finally, *L. nanum* **n**. **sp**. is also different from *L. gastroides*, because the new species is larger in length and has narrower eggs than *L. gastroides*.

**Heterophyidae Leiper, 1909**

***Pygidiopsis*** **Looss, 1907**

***Pygidiopsis noctilus* n**. **sp**.

Taxonomic summary

Type-host: *Noctilio leporinus* (Linnaeus) (Chiroptera: Noctilionidae).

Type-locality: El Remate (20°30’25.2’’N 90°23’03.0’’W) Calkiní, Campeche, Mexico.

Other localities: Ich Ha Lol Xaan (19°56’30.75’’N, 90°22’30.85’’W), Hampolol, Campeche, Mexico.

Prevalence and mean intensity: 83.3% (5/6 bats) and 11.2 from Ich Ha Lol Xaan, and 50% (2/4) and 45 from El Remate.

Material examined: Holotype: (CNHE 11742) paratypes: (CNHE 11451)

Representative DNA sequence: OP837308, MW332629

Synonym: *Pygidiopsis macrostumun* Travassos, 1928 in Panti-May et al. (2021) and Moguel-Chin et al. (2023).

ZooBank Life Science Identifier: 6316F333-6F61-4012-8519-CF74542262C7

Etymology: The specific name refers to the genus of the host, ﻿*Noctilio leporinus*, one of the few mammals in which natural *Pygidiopsis* infections have been recorded.

### Description

**Fig. 4:**
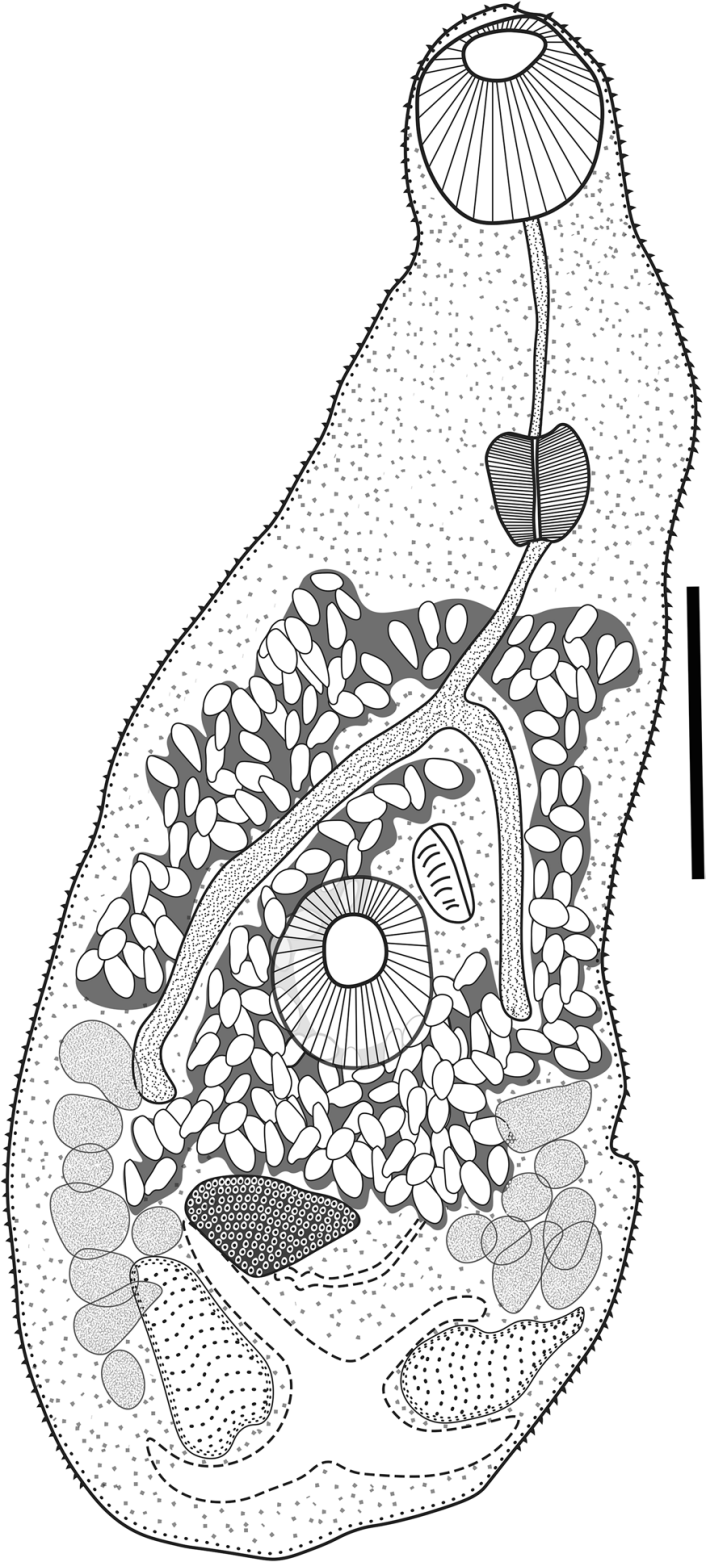
*Pygidiopsis noctilus* **n**. **sp**. ventral view of holotype. The scale bar=100 μm

**Fig. 5:**
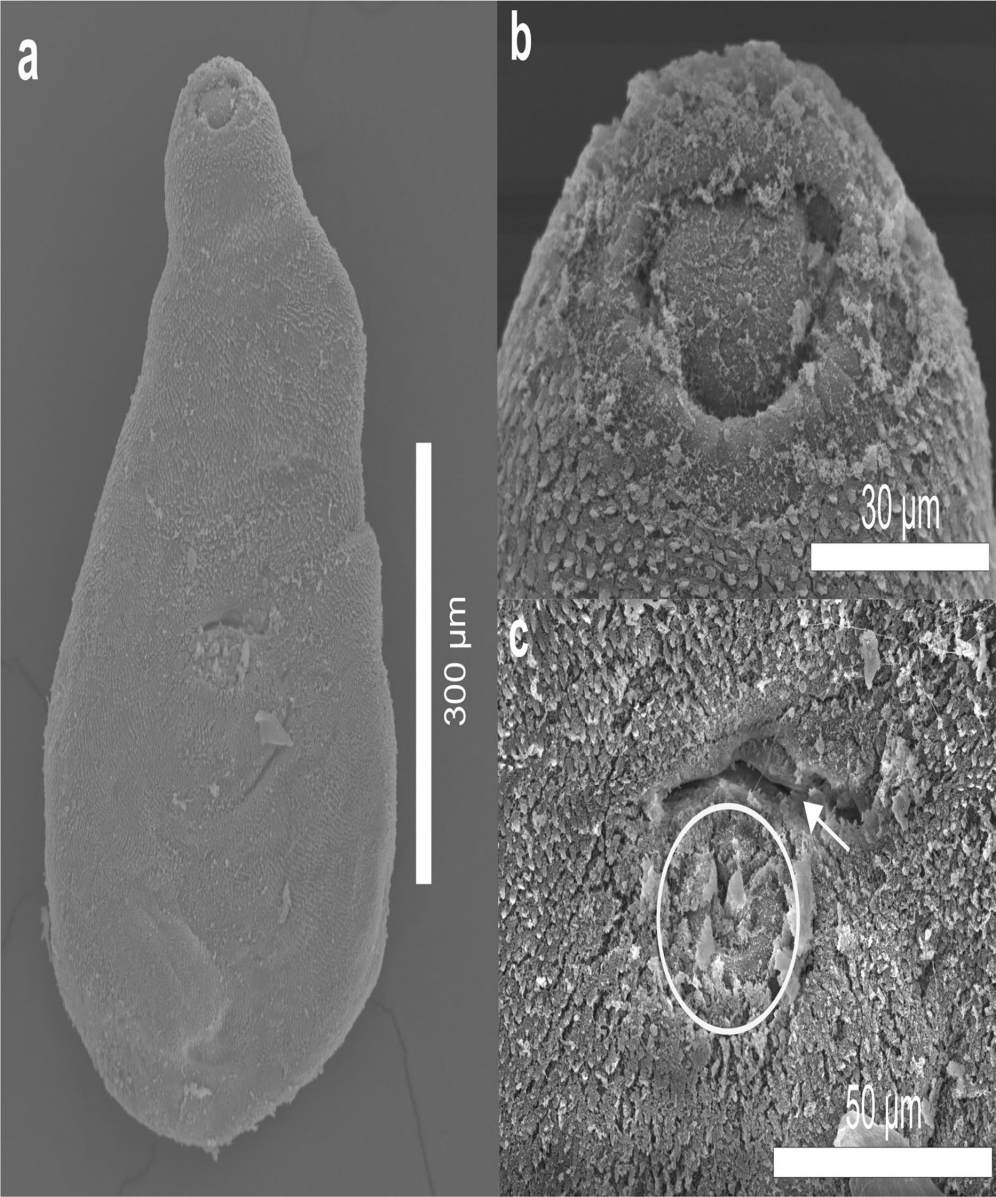
Scanning electron micrographs of *Pygidiopsis noctilius* **n**. **sp**. (a) general ventral view of the trematode. (b) oral sucker. (c) In the circle is the ventral sucker and the arrow point the genital pore

Based on 10 specimens (Fig. 4, Fig. 5). Body small and pyriform. Body length 460–695 (565) narrow at the ovarian level, width 190–265 (233.4). Tegument covered with regular and small spines extending along to the body. Oral sucker terminal 50–85 (67.4) × 60–90 (77.2). Ventral sucker muscular, median, in the second half of the body, slightly smaller than the oral sucker, 45–62 (55.2) × 50–69.7 (60.2). Ventral / oral sucker length ratio 1:0.5–0.9 (1:0.8). Ventral / oral sucker wide ratio 1:0.6–0.9 (1:0.7). Prepharynx extensible 52–82 (67.6) long. Pharynx muscular between oral sucker and the intestinal bifurcation 40–49 (45.7) × 32–45 (37.1). Oesophagous length 60–85 (72.5). Testes symmetrical, posterior to ventral sucker, slightly irregular in shape. Right testis 30–50 (41.2) × 45–82 (67.2). Left testis 40–52 (44.2) × 50–83 (66.5). The genital pore is anterior to the ventral sucker. Seminal vesicle oval, postero-sinistral to ventral sucker; ejaculatory duct opens into genital sac; genital sac antero-sinistral to ventral sucker. Ovary slightly irregular in shape, pre-testicular, 33–40 (37.4) × 50–70 (57.6). Vitellarium formed by small follicles in the hindbody, consisting of two non-confluent clusters, between the level of the testes and the ventral sucker. Uterus extended from the level of pharynx to the anterior testes zone. Eggs are operculate, 17–20 (18.5) × 9–10 (9.8) . X-shaped excretory vesicle with pore terminal (see Table 2).

**Table 2:** Morphometric measurements of the species of the genus *Pygidiopsis* described in this study

Specie	*P. noctilus* **n. sp.**	*P. noctilus* **n. sp.**	*P*. *crassus*	*P. macrostomum*	*P*. *himantopae*
Reference	Present study	Panti et al*.* 2021	Ostrowsky, 1995	Simões et al. 2005	Dronen et al. 2005
Type host	*Noctilio leporinus*	*Noctilio leporinus*	Laboratory mice	*Poecilia vivipara*	*Himantpus mexicanus*
Locaties	Campeche, Mexico	Campeche, Mexico	Buenos Aires, Argentina	Rio de Janeiro, Brazil	Rio de Janeiro, Brazil
Body length	512–658	460–695	378–588	739–1,020	475–650
Body width	240–250	190–265	294–386	354–477	245–445
Oral sucker	59.2–80 × 64–87	50–85 × 60–90	48–63 × 55–69	95–133 × 104–142	42–65 × 50–70
Ventral sucker	58.5–62 × 60–69.7	45–52 × 50–63	59–74 × 61–84	66–104 × 76–114	50–63 × 58–64
Prepharynx	52–82	NA	0–50	32–114	30–58
Pharynx	40–49 × 32–45	45–49 × 32–39	42–46 × 34–44	57–85 × 46–60	37–48 × 35–58
Oesophagus	60–85	NA	15–32	32–47	45–95
Ovary	33–37 × 52–70	40 × 50–55	38–50 × 63-84	76–104 × 85–114	45–80 × 55–88
Right testis	30–45.5 × 60–82	35–50 × 45–70	42–69 × 84–130	123–154 × 77–95	55–70 × 60–111
Left testis	42–45 × 59–83	40–52 × 50–75	42–67 × 84–116	123–154 × 77–95	45–100 × 70–138
Eggs	18–20 × 9 –10	17–19 × 9–10	18.9–23.1 × 10.5–12.6	21–23 × 11–14	17–20 × 8–12

### Remarks

*Pygidiopsis noctilus* **n**. **sp**. conforms to the diagnosis of *Pygidiopsis* as given by Looss (1907) by presenting some characteristics such as testes opposite, genital pore antero-sinistral to the ventral sucker and body of 400–700 long. The new species can be easily distinguished from 11 of the 14 congeneric species by having the vitellaria reached the posterior border of the ventral sucker and the uterus extended close to the pharynx. Three species share this character *P. macrostomum*, *P*. *crassus* Strowski, 1995 and *P. himantopae* Dronen, 2005. *Pygidiopsis noctilus* **n**. **sp**. can be readily distinguished from *P*. *himantopae* by having smaller testes (right testis 30–50 × 45–82, left testis 40–52 × 50–83 vs right testis 55–70 × 60–111, left testis 45–100 × 70–138) and ovary (33–40 × 50–70 vs. 45–80 × 55–88); additionally, the oral sucker is bigger than ventral sucker in *P. noctilus* **n**. **sp**. while in *P*. *himantopae* suckers are equal (1:0.5–0.9 vs 1:1). The new species can be differentiated from *P*. *crassus* in possessing the oral sucker bigger than the ventral sucker (1:0.5–0.9 × 1:0.6–0.9 vs 1:1.07–1.23 × 1: 1.1–1.2), and smaller eggs (17–20 × 9–10 vs 18.9–23.1 × 10.5–12.6). *Pygidiopsis noctilus* **n**. **sp**. differs from *P*. *macrostomum* because in the new species the oesophagous and the prepharynx are equal (60–85 and 52–82, respectively) whereas in *P*. *macrostomum* the oesophagous is shorter than the prepharynx (32–47 and 32–114, respectively); also the eggs are smaller (17–20 × 9–10 vs 21–23 × 11–14), the pharynx is more symmetrical (40–49 × 32–45 vs 57–85 × 46–60), and testes and ovary are smaller in the new species (see Table 2).

**Dicrocoeliidae Looss, 1899**

***Brachylecithum*** **Shtrom, 1940**

***Brachylecithum rileyi* n**. **comb**.

(Synonym: *Dicrocoelium rileyi*)

Taxonomic summary

Host: *Nyctinomops laticaudatus* (Chiroptera: Molossidae).

Locality: Calcehtok (20°33’02.5’’ N, 89°54’44.4’’W) Opichén, Yucatan, Mexico.

Prevalence and mean intensity: 16.7% (2/12 bats) and 3 from Calcehtok.

Material examined: Number Catalogue (CNHE 11746)

Representative DNA sequence: OP837309

Synonyms: *Brachylecithum* sp. in Moguel-Chin et al. (2023), *Dicrocoelium rileyi* in Caballero and Caballero (1969); Caspeta-Mandujano et al. (2017); Falcón-Ordaz et al. (2019); Guzmán-Cornejo et al. (2003); Martínez-Salazar et al. (2020).

ZooBank Life Science Identifier: D48C5F10-6371-4305-BF46-7A43284A3779

### Redescription

**Fig. 6:**
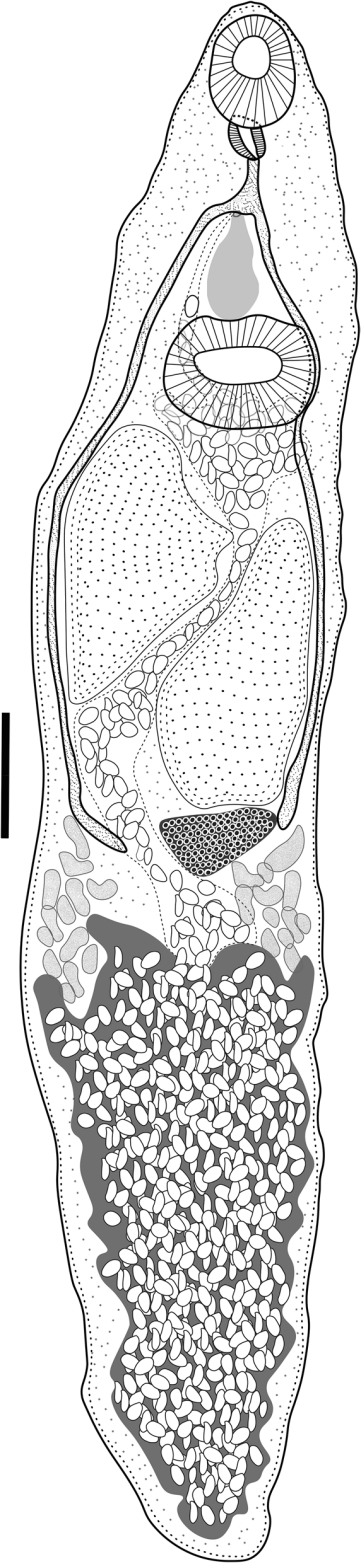
*Brachylecithum rileyi* **n**. **comb**. ventral view. The scale mesures 200 μm

Based on three adult specimens (Fig. 6). Body filiform, elongated, length 1550–2625 (1916.6) narrow in the forebody, width 294–431.2 (346.2), with tegument unspined. Oral sucker subterminal and oval 156.1–165.9 (161) × 136.6–151.2 (144.7). Ventral sucker in anterior third of body, round and bigger than oral sucker, 151.2–195.2 (172.4) × 146.4–209.8 (174). Ventral / oral sucker length ratio 1:0.9–1.2 (1:1.1). Ventral / oral sucker width/ ratio 1:1–1.3 (1:1.2). Pharynx muscular oval, 34.5–39.3 (37.6) × 29.2–53.6 (41.5). Oesophagous bifurcated between suckers. The caeca slender, extending to the ovary. Testes symmetrical, diagonal, asymmetrical and posterior to ventral sucker. Anterior testis 136.6–488 (255.3) × 219.6–292.8 (250.5). Posterior testis 146.4–519.4 (278.8) × 195.2–292.8 (244.3). Genital pore at the level of the bifurcation of the caeca. Cirrus-sac anterior to the ventral sucker, intercaecal, 126.8–200 (151.28) × 73.2–78 (76.4). The cirrus could not be observed. Ovary globular, post-testicular, smaller than testes, 78–82.9 (79.70) × 146.4–195.2 (167.5). Vitellarium follicular, not-confluent, in hindbody, post-testicular. Uterus covering the entire posterior part. Eggs numerous, 29.5–31.9 (30.3) × 17.2–19.6 (18.04).

### Remarks

*Brachylecithum rileyi* **n**. **comb**. is a species of Dicrocoeliidae that was originally described as *Dicrocoelium rileyi* by Macy (1931) who found it parasitizing the intestine of *Tadarida brasiliensis* (Le Conte) in Kansas, USA. Since then, it has been recorded in several places in Mexico in the same species of bat (Caballero and Caballero 1969; Guzmán-Cornejo et al., 2003; Caspeta-Mandujano et al., 2017; Falcón-Ordaz et al., 2019) and in *N*. *laticaudatus* (Moguel-Chin et al., 2023). When the species was described, the genus *Brachylecithum* had not yet been erected, which was formally described by Shtrom (1940). This author considered that the extension of the caeca and of the vitellarium were important characteristics to separate *Brachylecithum* from *Dicrocoelium*, because in *Brachylecithum* the caeca are short (terminate far from posterior extremity of body) and the vitellarium is composed of few large follicles and does not reach anteriorly to the ovary, while in *Dicrocoelium* the caeca are longer (reaching closer to the posterior extremity of the body) and the vitellarium is composed of small follicles that do reach the ovary and even in some species, the follicles extend beyond the anterior border (Pojmańska 2008). Therefore, due to the extension of the caeca and of the vitellarium in *B. rileyi* **n**. **comb**., this species agrees rather with the diagnosis of *Brachylecithum* and not with *Dicrocoelium*. Unfortunately, in more recent studies, where it can also be seen that the morphology of the specimens agrees with *Brachylecithum* (i.g. Caspeta-Mandujano et al. 2017; Falcón-Ordaz et al. 2019), the researchers did not note the diagnostic differences between these genera, and they continued to name their specimens as *D*. *rileyi*.

### Phylogenetic relationships and genetic distance

**Fig. 7:**
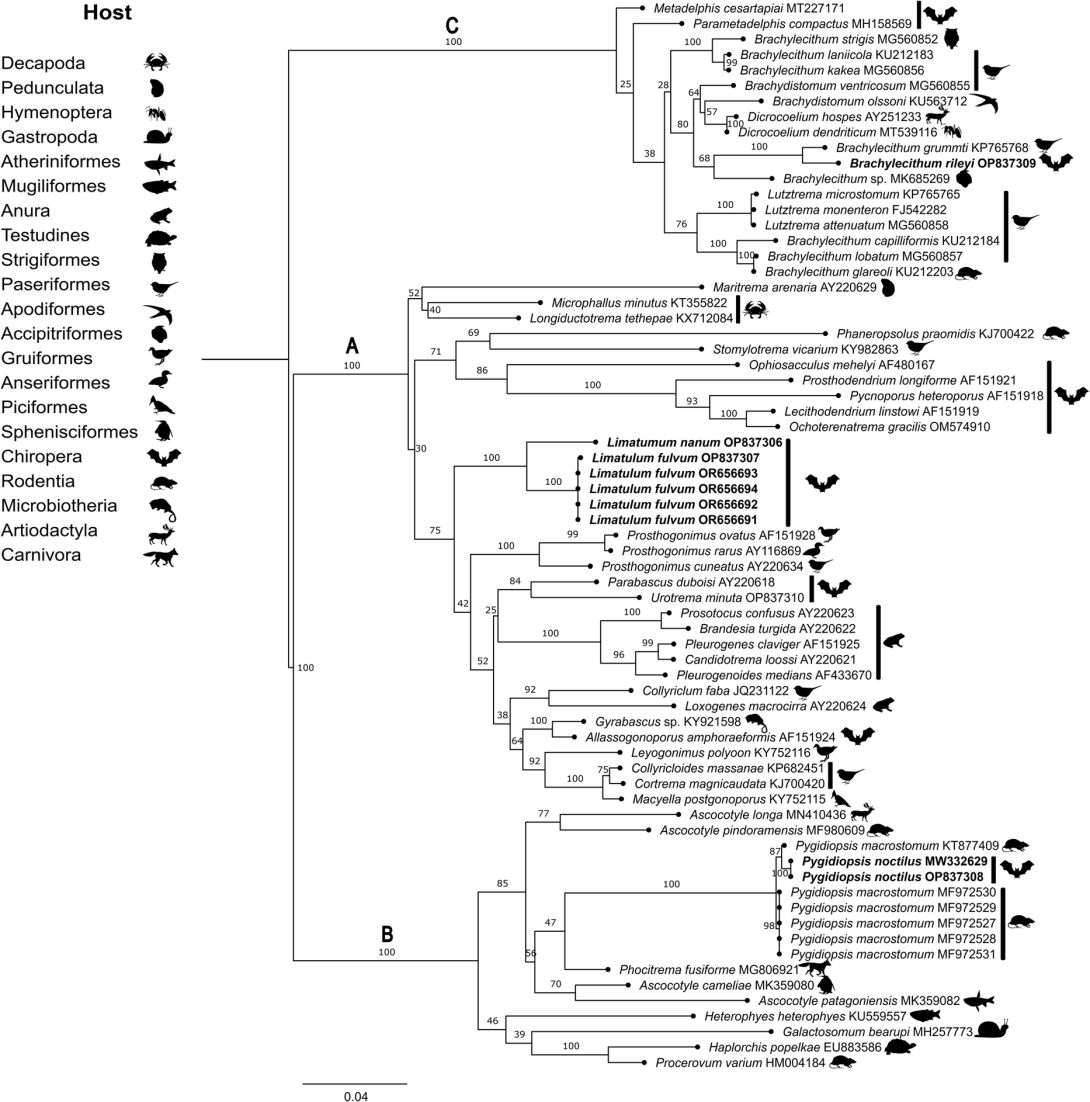
Phylogenetic tree based on the Maximum Likelihood analysis constructed on partial large subunit ribosomal gene (28S) of Trematoda species from different hosts (likelihood = -13616.961705). The sequences included in the analysis with * were obtained from larvae of the intermediate hosts. The sequences of the new species are in bold letters

The aligned data set for the 28S gene was of 1446 base pairs (bp) long and consisted of 69 sequences of Trematoda including sequences of *Brachylecithum rileyi* **n**. **comb**., *Pygidiopsis noctilus* **n**. **sp**., *Limatulum fulvum* **n**. **sp**. and *Limatulum nanum* **n**. **sp**. from Yucatan, Campeche and Veracruz, Mexico. The substitution model selected for this data set used to infer the ML phylogenetic hypothesis was GTR+CAT. The nucleotide frequencies were A= 0.215 C= 0.221 G= 0.318 T= 0.246. The ML tree had a value of ln = -13616.961705. Our specimens were grouped in three main clades (Fig. 7). The first clade named “A” grouped species of Lecithodendriidae, Stomylotrematidae, Prosthogonimidae, Pleurogenidae, Phaneropsolidae and Microphallidae (superfamily: Microphalloidea) (bootstrap = 100). Our new species of *Limatulum* were grouped in the same subclade as a sister group of Pleurogenidae (Bootstrap= 100). Our sequences of *L. fulvum* **n**. **sp**. from Yucatan and Veracruz presented low genetic differences of 0.09%. This species presented genetic differences of 3.8–4.1% with *L. nanum* **n**. **sp**. In the second clade named “B”, species of Heterophyidae (superfamily: Opisthorchioidea) were grouped with high support values (Bootstrap= 100) and the specimens of *P*. *noctilus* **n**. **sp**. were nested as a sister species of *P*. *macrostomum* (Bootstrap= 100). The sequences of *P. noctilus* **n**. **sp**. presented a null intraspecific genetic difference, but the genetic variation with the sequences of *P. macrostomum* from Brazil were of 0.5–0.6%. The third clade named “C” comprised members of the family Dicrocoeliidae (superfamily: Gorgoderoidea). In this clade, *Brachylecithum* was not monophyletic, and three subclades of this genus were obtained. Particularly, our sequence of *B*. *rileyi* **n**. **comb**. was grouped as sister species of *Brachylecithum grummti* Odening, 1963 from a bird of Brazil (bootstrap = 100). The genetic distance between these sister species was 2.1%.

## Discussion

The new *Limatulum* species described in this study are the ninth and tenth described for this genus in the Americas. On one hand, *L*. *fulvum* **n**. **sp**. is readily distinguishable from most congeners mainly by having: the smallest cirrus sac of all species in the genus, a long oesophagus, confluent vitelline glands that do not reach the level of the pharynx, and testes posterior to the ventral sucker. On the other hand, *L. nanum* **n**. **sp**. can be distinguishable from the other congeneric species because does not have a visible oesophagus, it has non-confluent vitelline glands in two lateral clusters and it has testes at the level of ventral sucker, in addition to the combination of several characteristics such as: the position of the genital pore, the size of the cirrus sac and the size of the eggs. These last characteristics are used for other authors to separate the species from the congeneric species (Brooks and Coggins 1983; Lunaschi et al., 2003). The 28S sequences of the new species represent the first DNA sequences for the genus *Limatulum*, which allowed us to explore the phylogenetic position of the genus. Phylogenetically, the new species are nested among themselves in a clade independent from other families of Microphalloidea. This result is interesting because *Limatulum* has been associated as part of the Phaneropsolidae (Lotz y Font 2008); however, in our phylogeny, *Phaneropsolus* is not grouped with *Limatulum*, indicating that both belong to different families. *Limatulum* has also been associated with Lecithodendriidae, but phylogenetically it does not group with species of this family either. However, the *Limatulum* clade is resolved as the sister group of the Pleurogenidae clade *sensu* Tkach et al. (2019). Information on the phylogenetic position of *Limatulum* indicates that an eventual detailed taxonomic review of the diagnoses of Phaneropsolidae, Lecithodendriidae and Pleurogenidae is necessary, since the limits between these families seem to be diffuse, which has led to confusion and movements within their classifications.

*Pygidiopsis noctilus* **n**. **sp**. is the first species of the genus described in Mexico and the fifteenth species of the genus. Natural infections of this genus in mammals are rare. This group of parasites are generally found in birds and fishes, and the few records in mammals (e.g. rodents) come from experimental infections (Ostrowski de Núñez 1995; Ostrowski de Núñez 1996; Sohn et al. 2016). *Pygidiopsis noctilus* **n**. **sp**. is easily distinguished from most congeners because it possesses vitelline glands that reach the posterior border of the ventral sucker and the uterus extends to the pharynx. These characteristics are mentioned by Ostrowski de Núnez (1995) to differentiate the species of the genus. However, *P*. *noctilus* **n**. **sp**. can also be diagnosed by the combination of some characters such as: the ventral / oral sucker length ratio, the size of eggs, ovary and testes. For *Pygidiopsis*, we found sequences only for one congeneric species, and the tree show that *P. noctilus* **n**. **sp**. and *P*. *macrostomum* are sister species. *Pygidiopsis macrostomum* was recorded in Brazil through experimental infections of *Rattus norvegicus* (Berkenhout) (Simões et al. 2005) and also *Pygidiopsis* sp. (cf. *macrostomum*) was found in Cuba in *N*. *leporinus* (the same host of the new species) (Odening 1969). In the databases we only found DNA sequences of *Pygidiopsis macrostomum* from Brazil, but not from Cuba. Due to the geographic distribution, the host affinity and the similarity of some diagnostic characteristics such as the size of the eggs (18-19 × 9-11 vs 18–20 × 9 –10 in *P*. *noctilus* **n**. **sp**.) of the *Pygidiopsis* from Cuba, we infer that it is *P*. *noctilus* **n**. **sp**. instead of *P*. *macrostomum*, but to corroborate it will be necessary to sequence the specimens from other geographical regions in the future. The few genetic distances between *P*. *noctilus* **n**. **sp**. and *P*. *macrostomum* may indicate a recent divergence between both species and a low substitution rate of the 28S region, which is why there are also some similarities in morphology. However, the geographic distribution, the phylogenetic grouping and the specific morphological differences between both species are evidence to confirm the existence of the new species.

Regarding *Brachylecithum*, only some species of this genus have been reported in mammals (Rodentia, Insectivora and Chyroptera) (Casanova y Ribas 2004). The new combination of *Brachylecithum rileyi* increases the number of records for the genus in mammals. When the morphology of this species is studied it may be obvious that it does not belong to *Dicrocoelium sensu stricto*, but we think that several factors could have influence the delay in recognizing *B*. *rileyi* **n**. **comb**., and we can mention at least two. On one hand, the species was described before *Brachylecithum* was erected, and because the original description was published in Russian, information did not flow so quickly to the Western world and consequently it continued to be erroneously named *D*. *rileyi* for all these years in a domino effect. On the other hand, the uterus may become saturated with eggs causing that the extension of the caeca and vitelline follicles could not be assessed successfully, and this could have confused the determination of the specimens at the genus level in previous works. In the clade “C” *Brachylecithum* form several subclades. The phylogeny of the family are poorly resolved and the relation between their genera were unclear, so some authors have suggested a systematic review of several genera of the family Dicrocoeliidae which may explain the non-monophyly of the genera (Tkach et al., 2001a, 2001b).

The identification bases only in the morphology may allow inaccurate identifications due to the natural variations in size, organ position and structure body shape according to intrinsic parasite factors (e.g. age) and the methods of collection and fixation (Aldhoun et al., 2018). This study points out the importance of comparing morphological and phylogenetic information. It is essencial to continue with the studies on parasites of bats, especially on members of families that lack records of helminths.

### Supplementary Information

Below is the link to the electronic supplementary material.

Supplementary file1 (DOCX 14 KB)

Supplementary file2 (DOCX 26 KB)
