# Supplementary material for: Description of new species of Trematoda from bats of Southeastern Mexico and a new classification for Brachylecithum rileyi n. comb. (Dicrocoeliidae)
Source: Syst Parasitol. 2023 Dec 18;101(1):4. doi: 10.1007/s11230-023-10127-y (PMC10725855; doi:10.1007/s11230-023-10127-y)
Supplement: Supplementary file 1 — Supplementary file1 (DOCX 14 KB) [file 11230_2023_10127_MOESM1_ESM.docx]

Supplementary table 1: Accession number of bat symbiotypes deposited at the Colección Zoológica, Universidad Autónoma de Yucatán Mexico.

| Bat species | Catalogue number |
| --- | --- |
| *Eumops nanus* | 1612 |
| *Nyctinomops laticaudatus* | 1474, 1476 |
| *Pteronotus fulvus* | 1469, 1470, 1473, 1480 |
| *Noctilio leporinus* | 1613, 1614, 1615 |
