# Supplementary material for: Description of new species of Trematoda from bats of Southeastern Mexico and a new classification for Brachylecithum rileyi n. comb. (Dicrocoeliidae)
Source: Syst Parasitol. 2023 Dec 18;101(1):4. doi: 10.1007/s11230-023-10127-y (PMC10725855; doi:10.1007/s11230-023-10127-y)
Supplement: Supplementary file 2 — Supplementary file2 (DOCX 26 KB) [file 11230_2023_10127_MOESM2_ESM.docx]

Supplementary table 2: listed of trematodes species used in this study including their hosts, geographical origin of material, genbank accession numbers.

| Helminth taxa | Host | Contry | Accession number | Reference |
| --- | --- | --- | --- | --- |
| *Brachilecithum rileyi* | *Nyctinomops laticaudatus* | Mexico | OP837309 | Moguel-Chin et al. (2023) |
| *Limatulum fulvum* | *Pteronotus fulvus* | Mexico | OR656691  OR656692  OR656693  OR656694 | Present study |
| *Limatulum fulvum* | *Pteronotus fulvus* | Mexico | OP837307 | Moguel-Chin et al. (2023) |
| *Limatulum* *nanum* | *Eumops nanus* | Mexico | OP837306 | Moguel-Chin et al. (2023) |
| *Pygidiopsis macrostomum* | *Noctilio leporinus* | Mexico | OP837308 | Moguel-Chin et al. (2023) |
| *Metadelphis cesartapiai* | *Anoura peruana* | Ecuador | MT227171 | Achatz et al. (2020) |
| *Parametadelphis compactus* | *Lonchophylla handleyi* | Peru | MH158569 | Tkach et al. (2018) |
| *Brachyletithum gummti* | *Attila cinnamomeus* | Brazil | KP765768 | Hildebrand et al. (2015) |
| *Brachylecithum* sp. | *Accipiter badius* | Pakistan | MK685269 | Suleman et al. (2020) |
| *Brachydistomum ventricosum* | *Erithacus rubecula* | Czech Republic | MG560855 | Aldhoun et al. (2018) |
| *Dicrocoelium hospes* | NA | NA | AY251233 | *Direct submission by Dittmar (2003) |
| *Dicrocoelium dedriticum* | NA | Iran | MT539116 | Ebrahimpour et al., unpublished |
| *Brachydistomum olssoni* | *Apus apus* | Czech Republic | KU563712 | Heneberg et al., unpublished |
| *Brachylecithum strigis* | *Otus scops* | Czech Republic | MG560852 | Aldhoun et al. (2018) |
| *Brachylecithum laniicola* | *Lanius collurio* | Czech Republic | KU212183 | Aldhoun et al. (2018) |
| *Brachylecithum kakea* | *Acrocephalus scirpaceus* | Czech Republic | MG560856 | Aldhoun et al. (2018) |
| *Brachylecithum capilliformis* | *Locustella fluviatilis* | Czech Republic | KU212184 | Aldhoun et al. (2018) |
| *Brachylecithum lobatum* | *Emberiza schoeniclus* | Czech Republic | MG560857 | Aldhoun et al. (2018) |
| *Brachylecithum glareoli* | *Myodes glareolus* | Poland | KU212203 | Hildebrand et al. (2016) |
| *Lutztrema monenteron* | *Turdus migratorius* | USA | FJ542282 | Kinsella and Tkach (2009) |
| *Lutztrema microstomum* | *Cyanocitta cristata* | USA | KP765765 | Hildebrand et al. (2015) |
| *Lutztrema attenuatum* | *Sturnus vulgaris* | Czech Republic | MG560858 | Aldhoun et al. (2018) |
| *Brandesia turgida* | *Rana lessonae* | Ukraine | AY220622 | Tkach et al. (2003) |
| *Prosotocus confusus* | *Rana lessonae* | Ukraine | AY220623 | Tkach et al. (2003) |
| *Pleurogenoides medians* | *Rana lessonae* | Ukraine | AF433670 | Tkach et al. (2001) |
| *Pleurogenes claviger* | *Rana temporaria* | Ukraine | AF151925 | Tkach et al. (2000) |
| *Candidotrema loossi* | *Rana ridibunda* | Ukraine | AY220621 | Tkach et al. (2003) |
| *Parabascus duboisi* | *Myotis daubentoni* | Ukraine | AY220618 | Tkach et al. (2003) |
| *Urotrema minuta* | *Nyctinomoops laticaudatus* | Mexico | OP837310 | Moguel-Chin et al. (2023) |
| *Ascocotyle longa* | *Steno bredanensis* | Brazil | MN410436 | *Direct submissión by Ebert (2019) |
| *Ascocotyle pindoramensis* | *Mesocricetus auratus* | Brazil | MF980609 | Santos and Borges (2020) |
| *Pygidiopsis macrostomum* | *Mesocricetus auratus* | Brazil | KT877409 | *Direct submission by Borges et al. (2015) |
| *Pygidiopsis macrostomum* | *Noctilio leporinus* | Mexico | MW332629 | Panti-May et al., (2021) |
| *Pygidiopsis macrostomum* | *Mesocricetus auratus* | Brazil | MF972530 | Santos and Borges (2020) |
| *Pygidiopsis macrostomum* | *Mesocricetus auratus* | Brazil | MF972529 | Santos and Borges (2020) |
| *Pygidiopsis macrostomum* | *Mesocricetus auratus* | Brazil | MF972527 | Santos and Borges (2020) |
| *Pygidiopsis macrostomum* | *Mesocricetus auratus* | Brazil | MF972528 | Santos and Borges (2020) |
| *Pygidiopsis macrostomum* | *Mesocricetus auratus* | Brazil | MF972531 | Santos and Borges (2020) |
| *Phocitrema fusiforme* | *Callorhinus ursinus* | USA | MG806921 | Kuzmina et al. (2018) |
| *Ascocotyle cameliae* | *Spheniscus magellanicus* | Argentina | MK359080 | Hernández-Orts et al. (2019) |
| *Ascocotyle patagoniensis* | *Odontesthes argentinensis* | Argentina | MK359082 | Hernández-Orts et al. (2019) |
| *Heterophyes heterophyes* | *Liza ramada* | Italy | KU559557 | Masala et al. (2016) |
| *Galactosomum bearupi* | *Clypeomorus batillariaeformis* | Australia | MH257773 | Huston et al. (2018) |
| *Haplorchis popelkae* | *Elseya dentata* | Australia | EU883586 | Snyder and Tkach (2009) |
| *Procerovum varium* | *Mesocricetus auratus* | Thailand | HM004184 | Thaenkham et al. (2010) |
| *Leyogonimus polyoon* | *Fulica atra* | Poland | KY752116 | Kanarek et al. (2017) |
| *Cortrema magnicaudata* | *Hirundo rustica* | Czech Republic | KJ700420 | Kanarek et al. (2014) |
| *Collyricloides massanae* | *Erithacus rubecula* | Czech Republic | KP682451 | Kanarek et al. (2015) |
| *Macyella postgonoporus* | *Dendrocopus major* | Czech Republic | KY752115 | Kanarek et al. (2017) |
| *Allassogonoporus amphoraeformis* | *Pipistrellus kuhli* | Ukraine | AF151924 | Tkach et al. (2000) |
| *Gyrabascus* sp. | *Dromiciops bozinovici* | Chile | KY921598 | Bell et al. (2018) |
| *Collyriclum faba* | *Saxicola rubetra* | Czech Republic | JQ231122 | Heneberg and Literák (2013) |
| *Langeronia macrocirra* | *Rana berlandieri* | Guatemala | AY220624 | Tkach et al. (2003) |
| *Prosthogonimus rarus* | *Anas querquedula* | Ukraine | AY116869 | Tkach et al. (2003) |
| *Prosthogonimus ovatus* | *Pica pica* | Ukraine | AF151928 | Tkach et al. (2000) |
| *Prosthogonimus cuneatus* | *Sturnus vulgaris* | Ukraine | AY220634 | Tkach et al. (2003) |
| *Ophiosacculus mehelyi* | *Eptesicus serotinus* | Ukraine | AF480167 | *Direct submission by Tkach (2002) |
| *8Phaneropsolus praomidis* | *Rhabdomys* sp. | Malawi | KJ700422 | Kanarek et al. (2014) |
| *Stomylotrema vicarium* | *Sclerurus mexicanus* | Peru | KY982863 | Kanarek et al. (2017) |
| *Paralecithodendrium longiforme* | *Myotis daubentonii* | Ukraine | AF151921 | Tkach et al. (2000) |
| *Lecithodendrium linstowi* | *Nyctalus noctula* | Ukraine | AF151919 | Tkach et al. (2000) |
| *Ochoterenatrema gracilis* | *Perimyotis subflavus* | USA | OM574910 | Fernandes et al. (2022) |
| *Pycnoporus heteroporus* | *Pipistrellus kuhli* | Ukraine | AF151918 | Tkach et al. (2000) |
| *Microphallus minutus* | *Cherax dispar* | Australia | KT355822 | Kudlai et al. (2015) |
| *Maritrema arenaria* | Pedunculata | UK | AY220629 | Tkach et al. (2003) |
| *Longiductotrema tethepae* | *Grapsus albolineatus* | Australia | KX712084 | Kudlai et al. (2016) |

NA: Not available

*: This sequence do not have an associated article
